# Rapid Molecular Detection of Multidrug-Resistant Tuberculosis by PCR-Nucleic Acid Lateral Flow Immunoassay

**DOI:** 10.1371/journal.pone.0137791

**Published:** 2015-09-10

**Authors:** Hatairat Kamphee, Angkana Chaiprasert, Therdsak Prammananan, Natpapas Wiriyachaiporn, Airin Kanchanatavee, Tararaj Dharakul

**Affiliations:** 1 Department of Immunology, Faculty of Medicine Siriraj Hospital, Mahidol University, Bangkok, Thailand; 2 Department of Microbiology, Faculty of Medicine Siriraj Hospital, Mahidol University, Bangkok, Thailand; 3 National Center for Genetic Engineering and Biotechnology, National Science and Technology Development Agency, Thailand Science Park, Pathumthani, Thailand; 4 National Nanotechnology Center, National Science and Technology Development Agency, Thailand Science Park, Pathumthani, Thailand; The Catholic University of the Sacred Heart, Rome, ITALY

## Abstract

Several existing molecular tests for multidrug-resistant tuberculosis (MDR-TB) are limited by complexity and cost, hindering their widespread application. The objective of this proof of concept study was to develop a simple Nucleic Acid Lateral Flow (NALF) immunoassay as a potential diagnostic alternative, to complement conventional PCR, for the rapid molecular detection of MDR-TB. The NALF device was designed using antibodies for the indirect detection of labeled PCR amplification products. Multiplex PCR was optimized to permit the simultaneous detection of the drug resistant determining mutations in the 81-bp hot spot region of the *rpoB* gene (rifampicin resistance), while semi-nested PCR was optimized for the S315T mutation detection in the *katG* gene (isoniazid resistance). The amplification process additionally targeted a conserved region of the genes as *Mycobacterium tuberculosis* (Mtb) DNA control. The optimized conditions were validated with the H37Rv wild-type (WT) Mtb isolate and Mtb isolates with known mutations (MT) within the *rpoB* and *katG* genes. Results indicate the correct identification of WT (drug susceptible) and MT (drug resistant) Mtb isolates, with the least limit of detection (LOD) being 10^4^ genomic copies per PCR reaction. NALF is a simple, rapid and low-cost device suitable for low resource settings where conventional PCR is already employed on a regular basis. Moreover, the use of antibody-based NALF to target primer-labels, without the requirement for DNA hybridization, renders the device generic, which could easily be adapted for the molecular diagnosis of other infectious and non-infectious diseases requiring nucleic acid detection.

## Introduction

Multidrug-resistant tuberculosis (MDR-TB) is defined by the resistance of *Mycobacterium tuberculosis* (Mtb) to at least the two most potent antimicrobials against TB infection, rifampicin (RIF) and isoniazid (INH) [1]. According to the WHO drug resistant TB surveillance report of 2014, MDR-TB occurred in 3.5% of new TB cases and 20.5% in previously diagnosed TB cases, with the incidence of MDR-TB estimated to be 5% of the overall TB cases on a global scale [2]. Every year, at least half a million new cases continue to emerge, adding to the existing MDR-TB burden [2]. The traditional culture based drug susceptibility testing (DST) remains the primary diagnostic platform in most developing countries. The consequent diagnostic time-delay is a major cause of escalating incidence. The key to preventing further spread is early detection and treatment.

A range of molecular diagnostic methods have been introduced into developing countries through the endorsement of the WHO [3,4]; however, several limitations hamper their popularity. The foremost drawback to molecular tests such as real-time PCR is the associated expense. Even though technologies like Xpert MTB/RIF (Cepheid, USA), a real-time PCR based detection system first endorsed by the WHO in 2010 [3], is sensitive enough to detect MDR-TB in HIV infected patients [5–7], the widespread use is unaffordable. Other molecular tests are largely PCR based endpoint detection systems such as INNO-LiPA Rif (Innogenetics, Belgium) and GenotypeMTBDR*plus* (Hains Lifesciences, Germany) that are DNA based strip tests. The test strips are lined with a wide array of mutation specific detection probes [8], which complicates result presentation. This strip design feature may be suitable for epidemiological surveys, but potentially impedes their practical use in routine diagnostics.

The primary objective of this proof of concept study was to develop a molecular diagnostic alternative for MDR-TB, targeting low-resource and peripheral healthcare settings that already routinely perform nucleic acid amplification. The aim was to create a highly simple, rapid, and easy-to-use detection tool, and to optimize its compatibility with conventional thermocycling technology. This limits the requirement for additional expenditure on instruments. The detection device developed is a one-step antibody-based Nucleic Acid Lateral Flow (NALF) immunoassay designed for the selective detection of specifically labeled nucleic acid within a PCR amplicon mixture.

The target Mtb genes for the PCR-NALF test in this study are *rpoB*, with mutations conferring RIF resistance [9,10], and *katG*, with mutations conferring INH resistance [11]. *RpoB* and *katG* mutation detection has been divided into two separate assays. Multiplex PCR was optimized for the *rpoB* assay, allowing for a simultaneous detection of multiple RIF resistance determining codons (531, 526 and 516) within the *rpoB* gene. Site- and mutation-specific primers for *rpoB* were designed and combined into one single assay. At any one time, only the primer specific to the mutation type binds to the target, from the multitude of primers, to register RIF resistance. This design strategy is practical because a simultaneous occurrence of more than one drug resistance conferring mutation in a single gene is uncommon. For the *katG* assay, semi-nested PCR was optimized for the detection of a single mutation (S315T), to register INH resistance. Primers were designed and evaluated in this study for their performance in identifying mutant (MT) templates, corresponding to drug resistant Mtb isolates, and the H37Rv wild-type (WT) template, corresponding drug susceptible Mtb isolate. All synthesized primers were labeled with specific tags for a rapid and easy detection by NALF antibodies. Both *rpoB* and *katG* assays follow the same test protocols, including the same thermocycling conditions. All NALF results were compared with the results of agarose gel electrophoresis for laboratory evaluation.

## Materials and Methods

### Template Preparation

Genomic DNA extracts from Mtb isolates (Table 1) were obtained from the Drug Resistant Tuberculosis Fund Laboratory, Department of Microbiology, Faculty of Medicine Siriraj Hospital, Mahidol University, Thailand. Outer primers were designed, using NCBI Primer-BLAST [12], to obtain specific regions of the target genes; RpoB-OF’ (5’-CGCTGTTGGAAAACTTGTTC-3’), RpoB-OR’ (5’-CTCCAGGAAGGGAATCATCG-3’), and KatG-OF’ (5’-GGCGGACCTGATTGTTTTCG-3’), KatG-OR’ (5’-GAGACAGTCAATCCCGATGC-3’). The amplified products of the target genes were ligated with pGEM T-Easy Vector (Promega, USA) (Table 1) and incubated overnight at 4°C, as per the protocol provided by the manufacturer. Competent *E*. *coli* DH5-α cells were transformed with the ligated plasmids and then grown on LB agar culture plate containing ampicillin, IPTG, and X-gal at 37°C overnight (16–18 hrs) for blue/white selection. The white colonies were picked and inoculated in LB broth with ampicillin and incubated overnight (12–16 hrs) at 37°C, with vigorous shaking (250 rpm). The overnight grown bacterial culture was then used for extracting the plasmid DNA (pDNA), using a plasmid extraction mini-kit (Favorgen, Taiwan). The presence of inserted genes was confirmed using restriction enzyme *EcoR1*-HF and by DNA sequencing (First Base, Singapore). Stocks of plasmids in DH5-α *E*. *coli* cells were made in 50% glycerol and stored at -80°C. The stored *E*. *coli* cells were regrown in LB broth (ampicillin) and pDNA extracted for use (mini-kit, Favorgen). The concentrations of the extracted pDNA were measured using a spectrophotometer (NanoDrop 8000, Thermo Scientific, USA).

**Table 1:** Bacteria and plasmid; source and function in template preparation.

Material	Type	Gene	Codon	Mutation type	Mtb Strain	Source/Strain	Function
Mtb genomic DNA extracts with known mutations	bacteria	*rpoB*	516	(D) Asp to Val (V)	DS 10216, DS 3315, DS 6279, DS 4230	clinical isolates	for target gene amplification
Mtb genomic DNA extracts with known mutations	bacteria	*rpoB*	526	(H) His to Tyr (Y), (H) His to Arg (R), (H) His to Asp (D), (H) His to Leu (L)	DS 6308, DS 8417, DS 5904, DS 0502, DS 4224, DS 9442, DS 6646	clinical isolates	for target gene amplification
Mtb genomic DNA extracts with known mutations	bacteria	*rpoB*	531	(S) Ser to Leu (L)	DS 9469, DS 6354, DS 6088, DS 6000	clinical isolates	for target gene amplification
Mtb genomic DNA extracts with known mutations	bacteria	*katG*	315	(S) Ser to Thr (T)	DS 12791, DS 11964, DS 10477, DS 12168	clinical isolates	for target gene amplification
Mtb reference strain	bacteria	-	-	-	-	H37Rv	experimental control
pGEM T-Easy Vector	plasmid DNA	-	-	-	-	commercial	target gene cloning
*E*. *coli*	bacteria	genome	-	-	-	DH5-α	host cell

### PCR Amplification

Amplification of the target genes, *rpoB* (locus Rv0667) and *katG* (locus Rv1908c) [13,14], were performed in two separate assays using labeled primers (Table 2). For the *rpoB* assay, RpoB-IF’ and RpoB-IR’ (Table 2) were designed to amplify a conserved region within the *rpoB* gene as Mtb DNA control. A set of *rpoB* mutant primers (forward) (Table 2) [15], together with RpoB-IR’, were also designed for specific mutation detection to determine Mtb RIF resistance in the *rpoB* assay. For the *katG* assay, KatG-IF’ and KatG-IR’ (Table 2) were designed for the amplification of a conserved region within the *katG* gene as Mtb DNA control, together with 315T-F’ (*katG* mutant forward) and KatG-IR’ (Table 2), designed for the S315T mutation detection to determine Mtb INH resistance in the *katG* assay (Table 2). RpoB-IF’ and RpoB-IR’, as well as KatG-IF’ and KatG-IR’ have also been designed to recognize a conserved region within the *rpoB* and *katG* genes, within the Mycobacterium tuberculosis complex (MTBC). All primers were designed using NCBI Primer-BLAST [12]. For the *rpoB* assay, individual forward primers targeting *rpoB* codons 531, 526 and 516 were first experimented separately to evaluate their efficacy and stringency. The individual *rpoB* primers were then combined into one single assay for multiplex PCR, for the simultaneous detection of all three codons. For the *katG* assay, a single forward primer (315T-F’) targeting the S315T mutation was combined with KatG-IF’ and KatG-IR’ for semi-nested PCR. All tests were initially performed using pDNA, with a concentration of 10^8^ copies (1 ng) added per PCR reaction. The experimental conditions were optimized for primer concentrations and amplification parameters. The optimized conditions were validated with Mtb isolates with known mutations; for the *rpoB* gene, 4 isolates for codon 516, 6 for codon 526, and 4 for codon 531 were used for the validation of the *rpoB* assay, and for the *katG* gene, 4 isolates with S315T mutation were used for the validation of the *katG* assay (Table 1). The *rpoB* and the *katG* assays were also validated with the H37Rv WT Mtb isolate. For each PCR reaction, 10^5^ DNA copies (1 ng) of genomic DNA extracts from Mtb isolates were added. PCR was performed in 25 μl reaction mixtures containing commercial 10x PCR buffer (composition of 1x buffer; 7.5 mM Tris-HCl (pH 8.75), 25 mM KCl, 1 mM MgCl_2_), 100 μM dNTP mix, 1 U Taq DNA Polymerase (Geneaid, Taiwan), and 1 ng purified pDNA/ 1 ng Mtb genomic DNA as amplification template. *RpoB* and *katG* assays were subjected to the same thermocycling conditions; initial denaturation at 95°C for 5 min, followed by 5 cycles of 94°C for 1 min and 72°C for 1 min, then 30 cycles of 94°C for 1 min, 63°C for 1 min, and 72°C for 1 min, with a final extension at 72°C for 7 min (T-Professional Thermocycler, Biometra, Germany). For each experiment, the negative control (-C), which consisted of PCR reagents without the addition of templates (NO TEMPLATE), were included to test for possible background appearance or false positives.

**Table 2:** Primers used in PCR amplification for the detection of INH and RIF resistance.

Primer	Label-Sequence	Conc. (μM)	Product Length (bp)
***katG* primers (for the detection of INH resistance)**			
KatG-IF’	**DIG**-AGCGGTAAGCGGGATCTGGAGAA	0.25	630
KatG-IR’	**Biotin**-CATGTCTCGGTGGATCAGCTTGTA	0.25	
315T-F’	**FITC**-GTAAGGACGCGATCACCA**c**	0.1	335
***rpoB* primers (for the detection of RIF resistance)**			
RpoB-IF’	**DIG**-GGAGGCGATCACACCGCAGACGT	0.1	314
RpoB-IR’	**Biotin**-TTTCGATGAACCCGAACG	1	
**Single mutation (SM) primers**			
SM-516V-F’	**FITC**-CTGAGCCAATTCATGG**t**	1	235
SM-526Y-F’	**FITC**-GTCGGGGTTGACC**t**	1	203
SM-526D-F’	**FITC**-GTCGGGGTTGACC**g**	1	203
SM-526R-F’	**FITC**-GTCGGGGTTGACCC**g**	1	203
SM-526L-F’	**FITC**-GTCGGGGTTGACCC**t**	1	203
SM-531L-F’	**FITC**-ACAAGCGCCGACTGT**t**	1	189
**Double mutation (DM) primers** ^**a**^			
DM-516V-F’	**FITC**-CTGAGCCAATTCAT**t**G**t**	1	235
DM-526Y-F’	**FITC**-GTCGGGGTTGAC**at**A	1	203
DM-526D-F’	**FITC**-GTCGGGGTTGAC**ag**A	1	203
DM-526R-F’	**FITC**-GTCGGGGTTGAC**a**C**g**	1	203
DM-526L-F’	**FITC**-GTCGGGGTTGAC**a**C**t**	1	203
DM-531L-F’	**FITC**-ACAAGCGCCGACT**a**T**t**	1	189

Reference

^a^[15].

**Fig 1:**
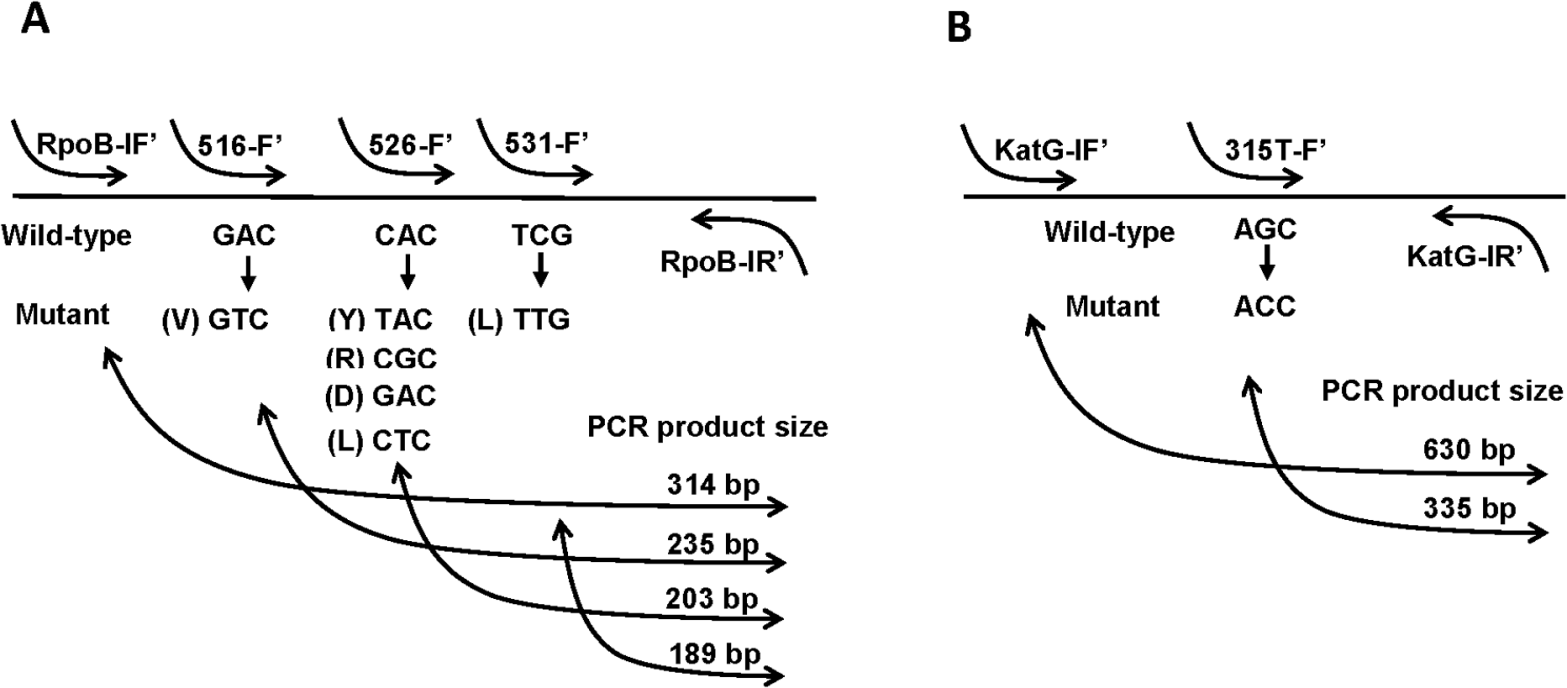
PCR Amplification Products. **(A)** Multiplex PCR amplification products for the *rpoB* assay and **(B)** Semi-nested PCR amplification products for the *katG* assay, on agarose gel electrophoresis.

For the *rpoB* assay, the FITC-labeled *rpoB* forward primers, together with the biotin-labeled RpoB-IR’ (Table 2), generate PCR products of 189 bp (codon 531), 203 bp (codon 526) and 235 bp (codon 516) in size (Fig 1). The DIG-labeled RpoB-IF’ (Table 2) and the biotin-labeled RpoB-IR’, generate PCR product of 314 bp in size (Fig 1). For the *katG* assay, the FITC-labeled 315T-F’ and the biotin-labeled KatG-IR’ (Table 2), generate PCR product of 335 bp (codon 315) in size (Fig 1). The DIG-labeled KatG-IF’ (Table 2), together with the biotin-labeled KatG-IR’, generate PCR product of 630 bp in size (Fig 1). With the use of the labeled primers, the PCR amplification products are rendered as dual labeled amplicons (either biotin-FITC, or biotin-DIG, on either end of the amplicon). After PCR amplification, 10 μl of PCR product were used for NALF detection. For analytical comparison of the results, 5 μl of PCR product were also used to run gel electrophoresis, and were visualized using 3% agarose gel (SeaKem, Lonza Inc., USA) infused with Gel-Red dye (Biotium, USA).

### Nucleic Acid Lateral Flow (NALF) Development

**Fig 2:**
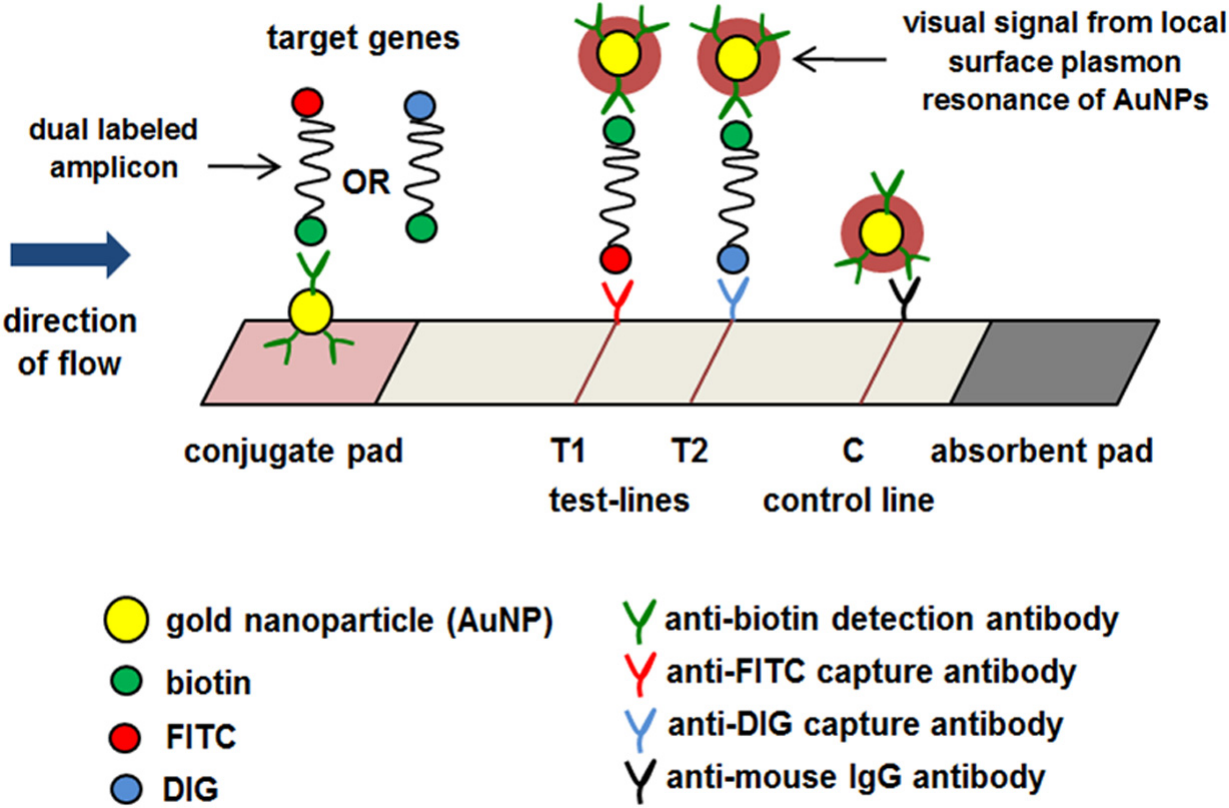
Schematic illustration of NALF design. The NALF device functions to indicate the presence of dual labeled amplicons in PCR products. At the conjugate pad, AuNP-anti-biotin binds to biotin on one end of the amplicon, and the complex flows towards the test-lines, T1 and T2. T1 is lined with anti-FITC mAb to capture biotin-FITC labeled amplicons, indicative of RIF resistance for the *rpoB* assay or INH resistance for the *katG* assay. T2 is lined with anti-DIG mAb to capture biotin-DIG labeled amplicons for Mtb DNA control. The excess AuNP-anti-biotin is captured at the control line (C) by anti-mouse IgG.

The NALF device has been designed for the indirect detection of amplified PCR products through the use of antibodies against primer-tags; FITC, DIG, and biotin (Fig 2). Monoclonal antibodies (mAbs) against each tag molecule were produced in-house using the mouse hybridoma technology, with specific recognition to FITC, DIG, and biotin (data not shown).

The NALF device consists of sample application pad, conjugate pad, nitrocellulose membrane and adsorption pad, which are assembled together in a plastic housing. NALF detection was performed individually for the *rpoB* and *katG* assays, using separate NALF devices for each assay. Following PCR, 10 μl of PCR product, either from the *rpoB* or the *katG* amplification assay, were mixed with 90 μl of NALF buffer solution and added to the NALF device at the sample pad. The mixture was then dispensed onto the conjugate pad, which hosts gold-nanoparticles (AuNPs) conjugated with anti-biotin mAbs (AuNP-anti-biotin) to detect dual labeled amplicons by binding with biotin (Fig 2). The complex flows along the nitrocellulose membrane towards the test-lines, T1 and T2.

**Fig 3:**
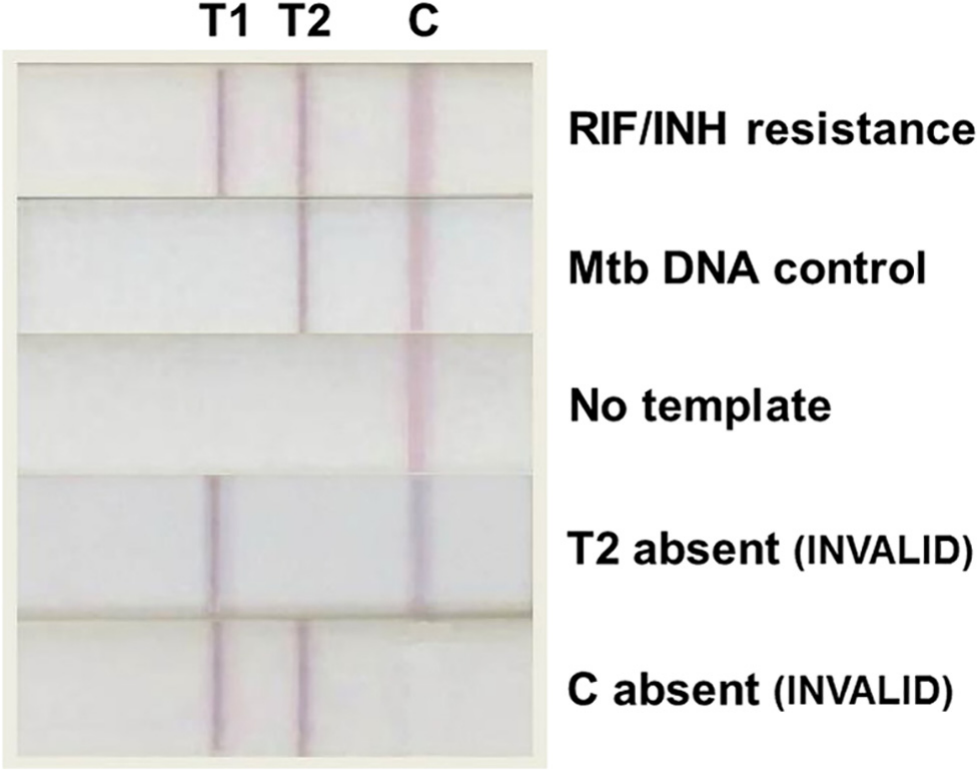
Example of possible NALF outcomes.

The first test-line (T1) is composed of anti-FITC mAb for capturing biotin-FITC-labeled amplicons to indicate RIF resistance in the *rpoB* assay or INH resistance in the *katG* assay (Fig 3); for *rpoB*, the appearance of T1 corresponds to PCR product sizes 189 bp, 203 bp or 235 bp on agarose gel, and for *katG*, to 335 bp on agarose gel (Fig 1). The second test-line (T2) hosts anti-DIG mAb to capture biotin-DIG-labeled amplicons for Mtb DNA control, which must appear in all cases for the results to be valid (Fig 3); for *rpoB*, the appearance of T2 correlates with 314 bp on agarose gel, and for *katG*, to 630 bp on agarose gel (Fig 1). The excess AuNPs-anti-biotin, unbound by amplicons, flow pass the two test-lines and are captured at the control line (C) by anti-mouse IgG to ensure the correct operation of the device. The absorbent pad functions as a wick to maintain the flow rate and direction, while preventing any back flow of fluid [16]. NALF results were read after 10 minutes of PCR product addition.

### Least Limit of Detection (LOD) Determination

The least limit of detection (LOD) is defined by the minimum number of copies of target genes required in each PCR reaction for a successful detection of the PCR products. The least limit of detection was determined using purified genomic DNA extracts from the H37Rv Mtb isolate, as well as Mtb isolates with known mutations (Table 1) as PCR amplification templates. To determine the LOD of the *rpoB* and *katG* assays, serial dilution of the templates was performed as follows; 10 ng (10^6^ copies of DNA), 1 ng (10^5^ copies of DNA), 0.1 ng (10^4^ copies of DNA), 0.01 ng (10^3^ copies of DNA), 0.001 ng (10^2^ copies of DNA). All NALF results were compared with the results of agarose gel electrophoresis for confirmation.

## Results

### The Selection of Genes and Mutation Sites for MDR-TB Determination

**Fig 4:**
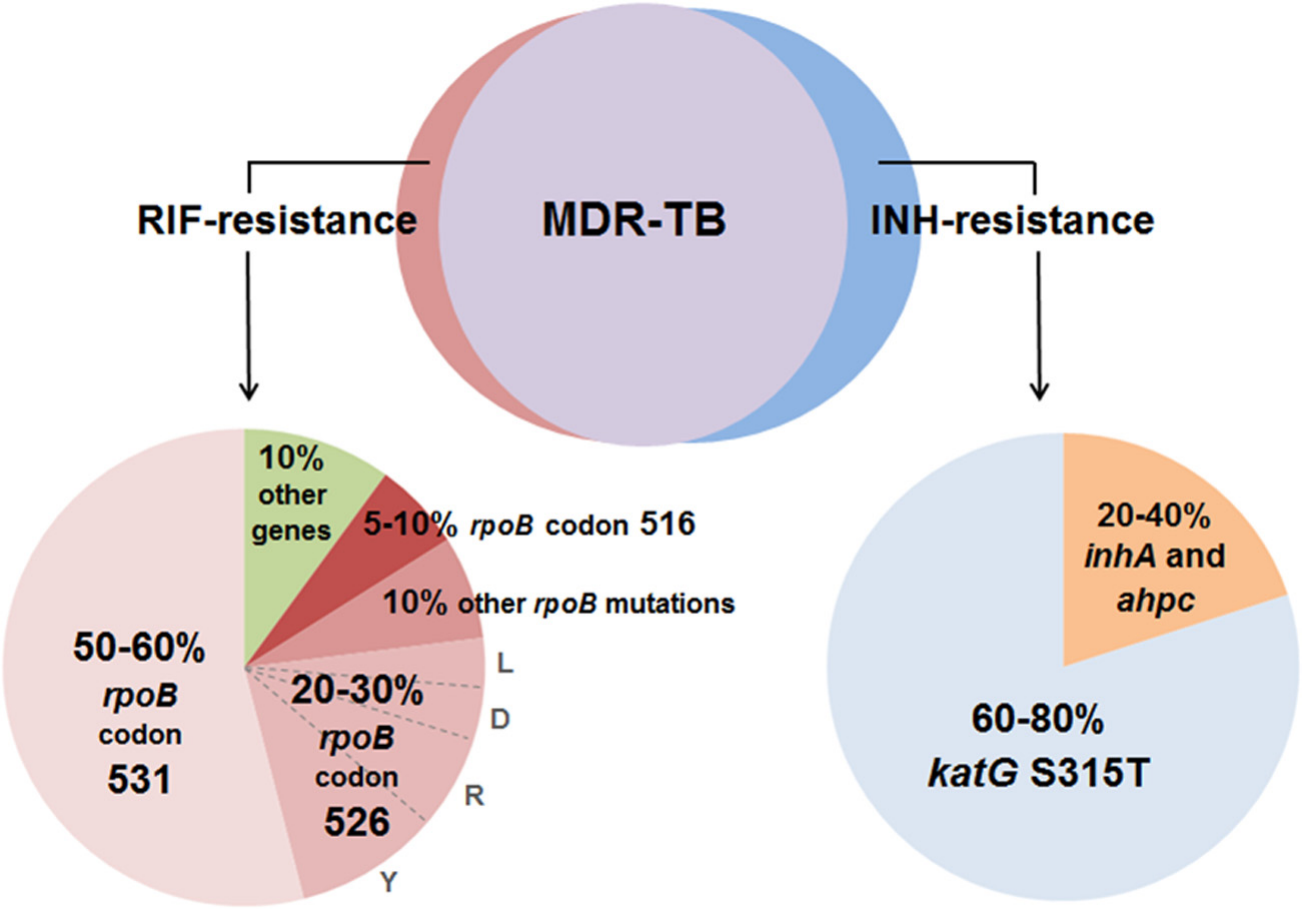
Distribution of MDR-TB determining mutations. A broad compilation of MDR-TB global epidemiological statistics represents the estimated percentage distribution of MDR-TB determining mutations. The compilation demonstrates that 85–95% of RIF resistance is accompanied by INH resistance to determine MDR-TB (purple). Five to fifteen percent of RIF resistance (brown crest) and 5–25% of INH resistance (blue crest) are monoresistance occurrences. [9–11,15,17,21–37,39–52].

In order to determine MDR-TB, this study focuses on the positive detection of RIF and INH resistance. Based on a broad compilation of MDR-TB global epidemiological statistics (Fig 4), 85–95% of RIF resistance has been found to naturally accompany INH resistance [17–23], making RIF resistance a widely used surrogate marker for MDR-TB; however, there is a 5–15% chance of RIF monoresistance development. 90–95% of RIF resistance confers mutation(s) in the 81-bp hot-spot region (codons 507–533) of the *rpoB* gene, called the rifampicin resistance-determining region (RRDR), with the highest global prevalence being at codons 531, 526 and 516. Multiple mutation types have been recorded per codon, with different statistical occurrence [24–28]. On the other hand, 60–80% of INH resistance is due to a single mutation (S315T) in the *katG* gene at codon 315 [29–34], with the rest occurring in other genes, such as in the promoter region of *inhA* and in *ahpC* genes [35–37]. INH resistance, however, is not commonly used as an independent marker for MDR-TB [38–42]. From the compiled data, the target mutation sites selected for MDR-TB determination in this study are *rpoB* codons 531, 526 and 516 for the *rpoB* assay in the determination of RIF resistance, with one mutation type of interest for codon 531 (L), four for codon 526 (Y, R, D and L), and one for codon 516 (V) (Table 1), with the highest statistical prevalence worldwide [17,18,23–28,43–47]. As for the *katG* assay, *katG* codon 315, with the S315T mutation, has been selected as the single mutation site in the determination of INH resistance [17–21,29–36,48–52].

### *KatG* PCR Amplification and Detection by NALF

**Fig 5:**
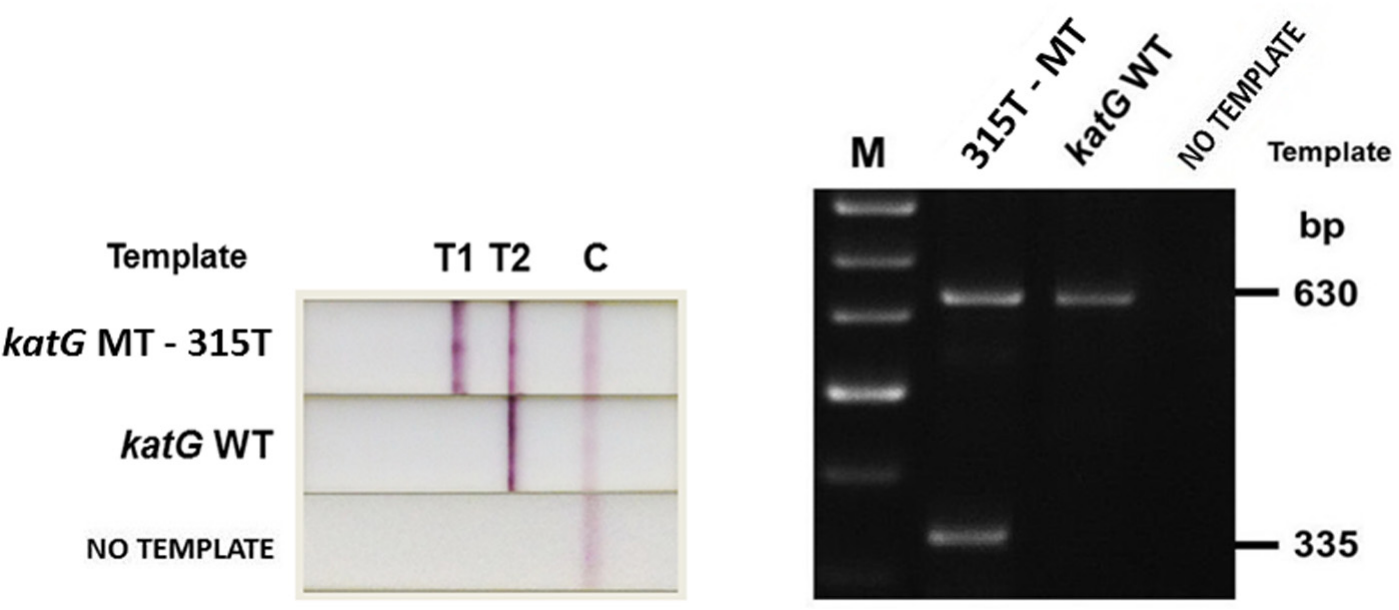
NALF and agarose gel electrophoresis results for the *katG* assay. NALF; the appearance of T1 and T2 for *katG* MT template indicates a positive detection of INH resistant Mtb isolate, whereas the appearance of T2 (only) for WT template indicates a positive detection of an INH susceptible Mtb isolate. Correspondingly, for gel electrophoresis, two bands are generated for MT template (630 bp, equivalent to NALF T2, and 335 bp to NALF T1), and a single band for WT template (630 bp, equivalent to NALF T2).

The determination of INH resistance in this study targets a single mutation (S315T) in the *katG* gene. Because the target mutation is singular, the use of just one mutation determining primer, 315T-F’ (Table 2), in the *katG* assay should be adequate. The functionality of *katG* primers and the ability to identify WT and MT templates were evaluated with the H37Rv Mtb isolate, along with four Mtb isolates containing the S315T mutation, via semi-nested PCR. The amplicons were detected using the NALF device and confirmed with agarose gel electrophoresis. The NALF results and the corresponding agarose gel electrophoresis results for all four Mtb isolates indicate the correct identification of MT and WT templates. Fig 5 is a representation of the results.

### Primer Design for the *rpoB* Assay

**Fig 6:**
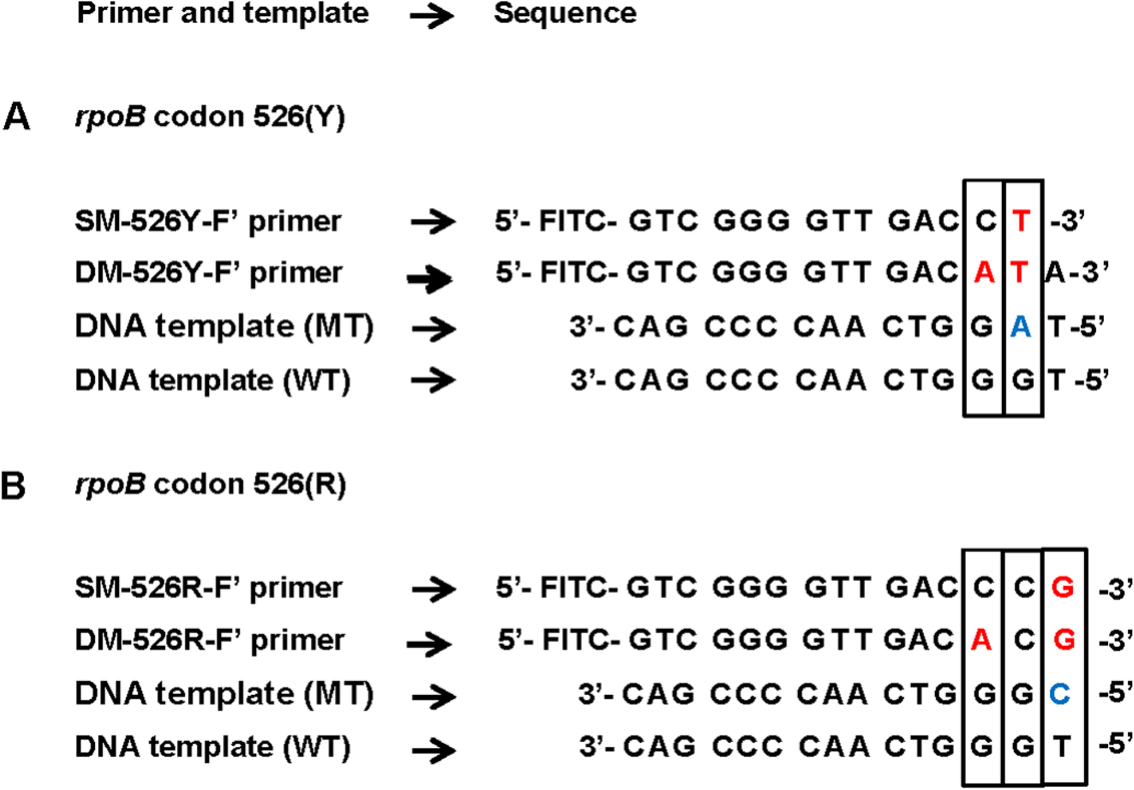
*RpoB* primer design strategy. *RpoB* primer designs for codon 526 (mutations Y and R) have been demonstrated above as examples. SM (single mutation) primers are designed with a single base mismatch at the 3’-terminus to complement MT templates. DM (double mutation) primers, which have also been designed to complement MT templates, carry an added mismatch at the third position from the 3’-terminus. The red colored bases indicate an intentional mismatch and the blue colored bases indicate a complementary base to the SM and DM primers.

*RpoB*, unlike *katG*, carries multiple mutation sites. To simultaneously detect all the target sites in one PCR reaction, the incorporation of several site- and mutation-specific primers into a single assay is required. The standard primer design of a single base mismatch between primer and non-target template, especially for codon 526 with four different target mutation types (Table 1), may not be enough to ensure correct template distinction. Therefore, the Yaku-Bonczyk primer design method, entailing an intentionally incorporated additional mismatch, has been explored in the study [15,53]. The added mismatch is expected to enhance specificity, leading to a better discrimination against non-complementary DNA. For the eventual selection of *rpoB* primers, for mutation detection within the *rpoB* assay, two primer sets were designed and assessed against each other; SM (single mutation) primers with the standard design of a single base mismatch at the 3’-terminus to complement MT templates, and DM (double mutation) primers with an additional strategically located mismatch at the third position from the 3’-terminus in accordance with the Yaku-Bonczyk method (Fig 6, Table 2).

### Mutant Primer Selection for the *rpoB* Assay

**Fig 7:**
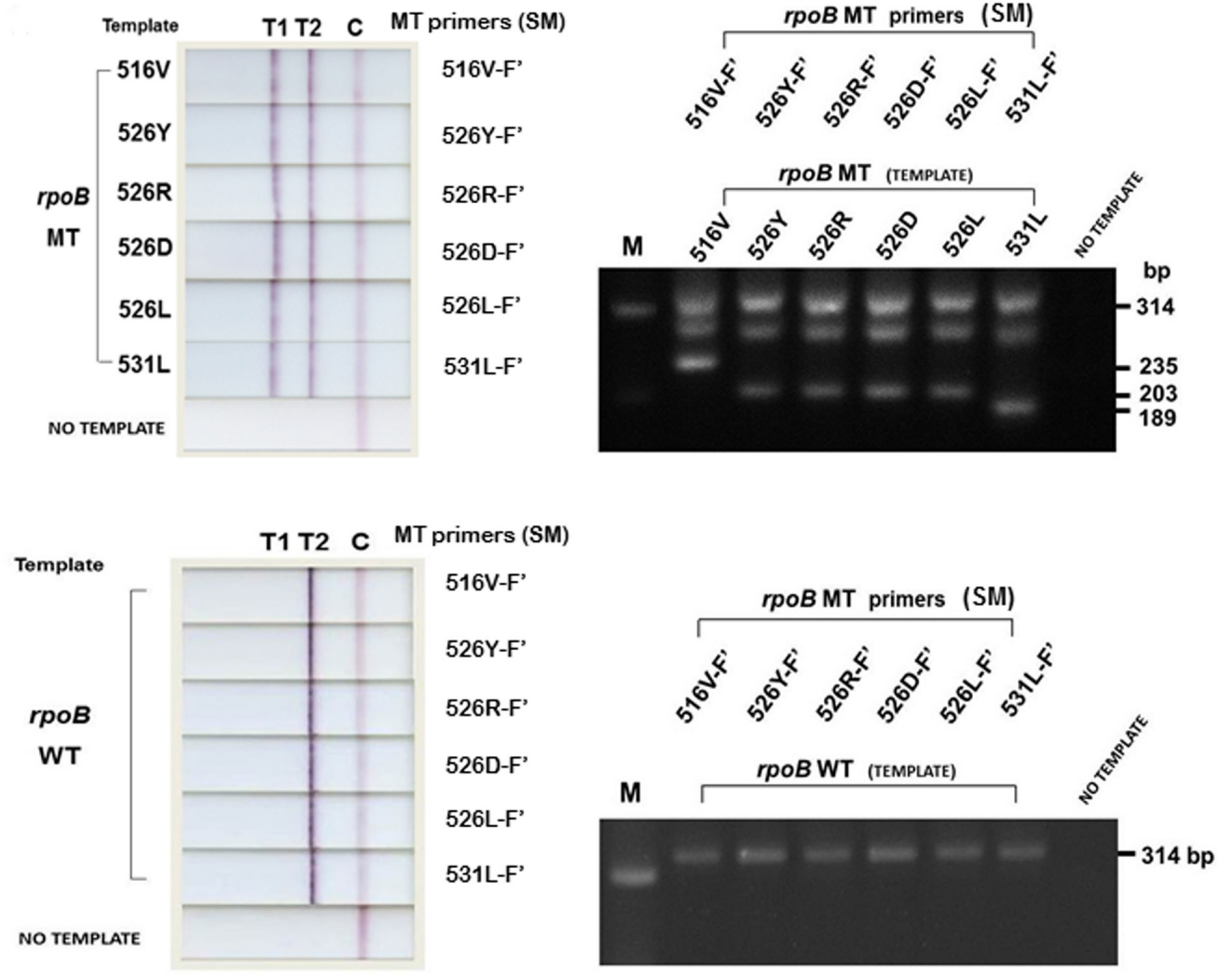
NALF and agarose gel electrophoresis results for the *rpoB* assay. **(A)** The use of SM primers demonstrate an effective template distinction with the appearance of NALF T1 and T2 for all MT templates to indicate RIF resistant Mtb isolates, which relates to the inner and outer DNA bands on agarose gel for the MT templates. As for WT templates, the assays correctly resulted in the appearance of NALF T2 only, to indicate RIF susceptible Mtb isolates, which corresponds to PCR product size 314 bp on agarose gel.

The SM and DM primers were tested with MT and WT templates in semi-nested PCR. Each primer was tested in a separate assay composed of RpoB-IF’, RpoB-IR’, and the target primer of interest for mutation detection (Table 2). The test results (Fig 7) show that SM primers were able to anneal to their specific targets, and were able to correctly identify the MT and WT templates with high efficacy. The appearance of an unaccountable non-specific band of approximately 270 bp on agarose gel in Fig 7 did not lead to a false positive/negative or background on NALF, and therefore, did not interfere with the test. DM primers, on the other hand, did not perform as expected. They failed to anneal to their targets, providing negative results (data not shown). DM primers were, therefore, exempted from further experimentation.

### Multiplex PCR for the Simultaneous Detection of RIF Resistance Determining Codons in the *rpoB* Gene

**Fig 8:**
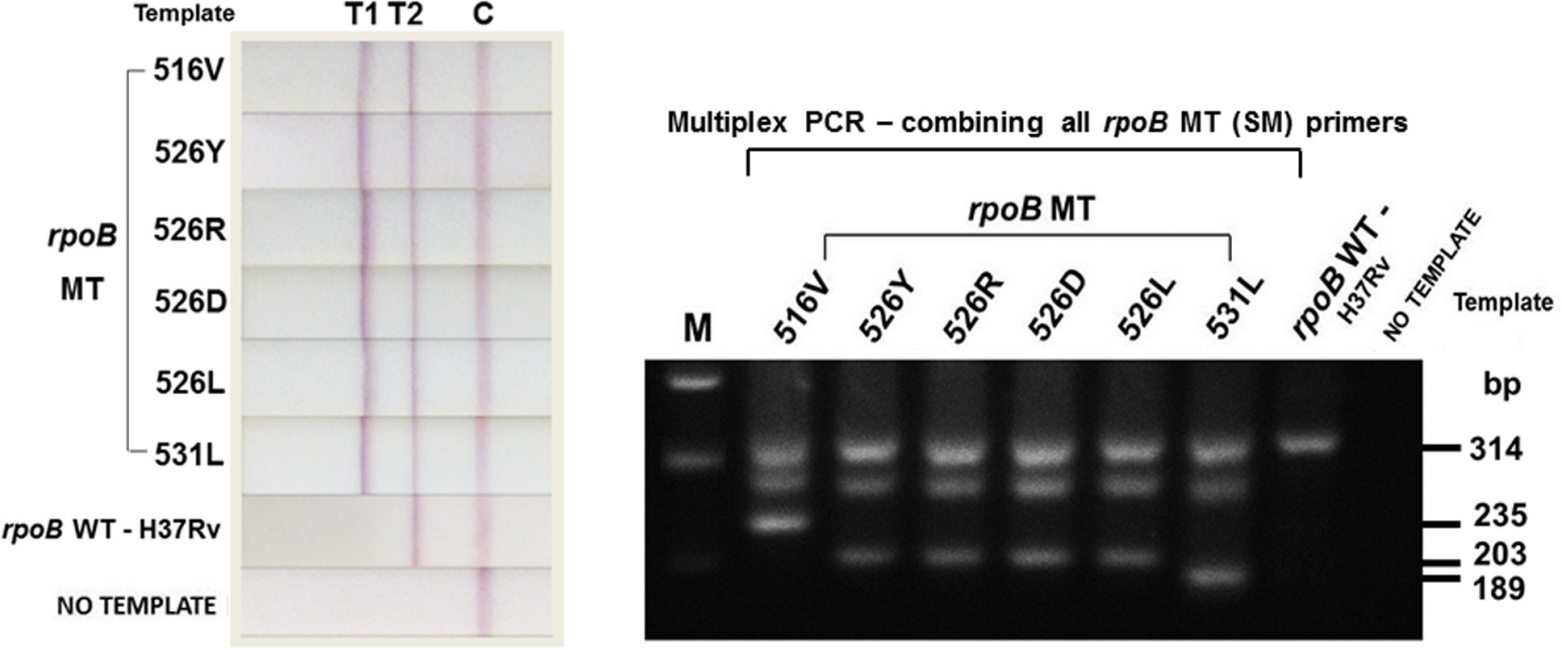
*RpoB* assay in multiplex PCR. NALF results for multiplex PCR presented with the correct appearance of T1 and T2 for MT templates to register RIF resistance Mtb isolates, and the appearance of just T2 for WT template to register RIF susceptible Mtb isolates.

SM primers, which have been selected for the *rpoB* assay, were combined into a single assay for multiplex PCR, which would allow all RIF resistance determining mutations at target codons 531, 526 and 516 to be detected simultaneously. Initially, the combined assay was tested using pDNA for condition optimization (data not shown). The optimized conditions were then validated and tested for reproducibility using the H37Rv (WT) Mtb isolate and Mtb isolates with known mutations (4 isolates for codon 516, 6 for codon 526, and 4 for codon 531) (Table 1). Fig 8 is a representation of the results, which reaffirms the efficacy, stringency and the specificity of the SM primers in the *rpoB* assay.

### Least Limit of Detection (LOD) Determination

**Fig 9:**
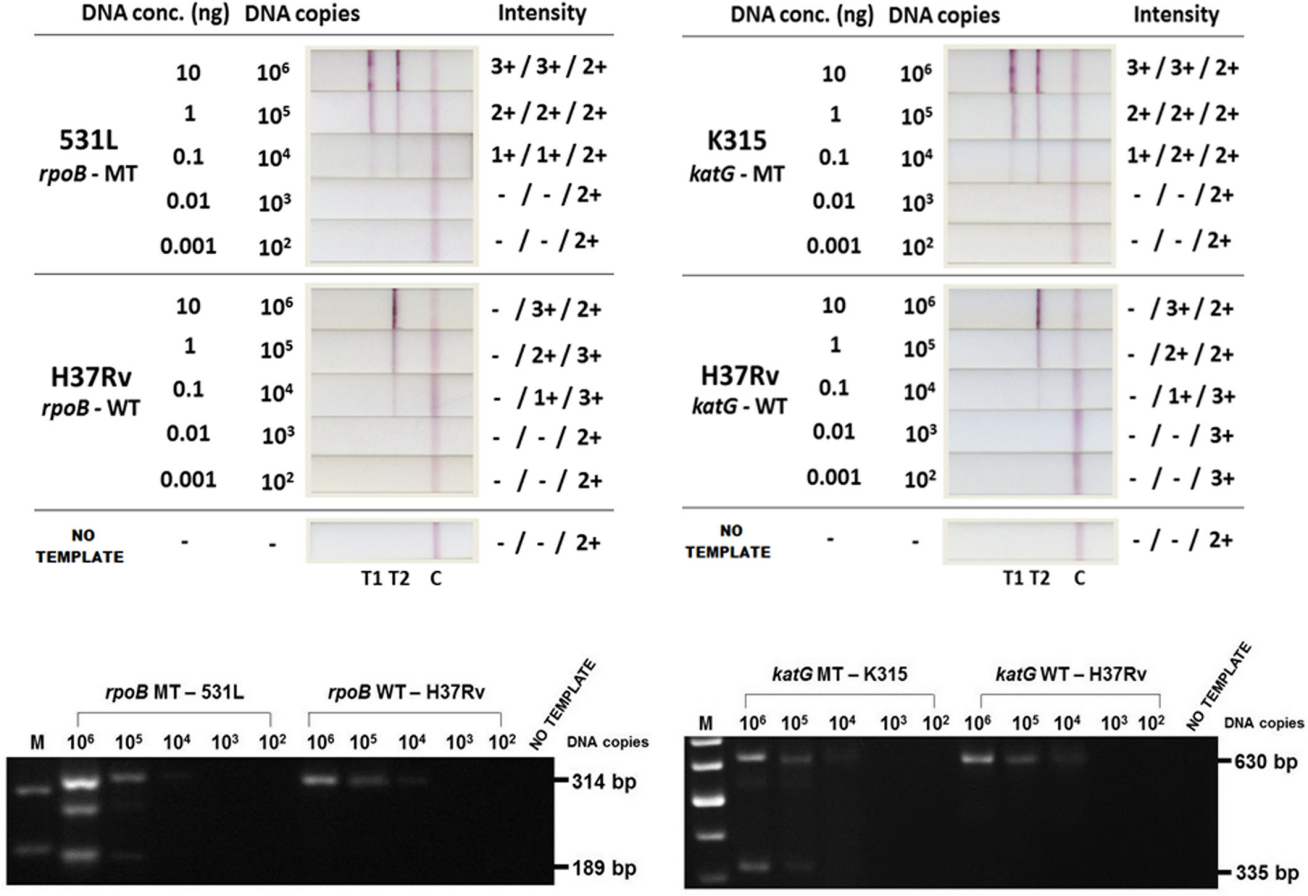
The least limit of detection (LOD); For both the *rpoB* and the *katG* assays, the NALF results present the LOD as 104 DNA copies (0.1 ng) per PCR reaction, whereas the gel electrophoresis results present the LOD as105 DNA copies (1 ng) per PCR reaction. Below these DNA concentrations, the NALF test lines or the agarose gel DNA bands are not observable. The LOD results for *rpoB* codon 531 have been shown above to represent the results for other *rpoB* codons.

The least limit of detection (LOD), which has been described in the study as the minimum number of copies of target genes required in each PCR reaction for a successful detection of the PCR products, was determined for both the *rpoB* (using SM primers) and the *katG* assays. The results indicate the LOD to be 10^4^ copies of DNA (0.1 ng) per PCR reaction via NALF detection, and 10^5^ copies of DNA (1 ng) per PCR reaction via detection by gel electrophoresis (Fig 9). The NALF test-lines illustrate a clear loss of intensity as the concentration of template DNA in the reactions diminished. Below the concentration of 10^4^ copies of DNA, the NALF test-lines were no longer visible. A lower LOD determined for NALF, compared to agarose gel electrophoresis, suggests a higher sensitivity of the device.

## Discussion

NALF technology is an interesting tool for enabling simplification of molecular diagnosis. Recent studies have successfully field-tested NALF for point-of-care molecular detection of malaria with promising results [54–56]. Preliminary tests have also been conducted for a lateral flow based quantitative detection of amplified HIV-RNA with clinical relevancy [57]. This proof of concept study, using the above principle, attempted to develop a molecular diagnostic alternative for MDR-TB to complement the use of conventional PCR, targeting low-resource and peripheral healthcare settings that already routinely employ PCR machines. The development of NALF, to meet the objective, was fixated upon simplicity and rapidity. These qualities were achieved through the strategic design to minimize operator involvement, and to present the results in the most non-complex manner. The approach uses gold nanoparticle (AuNP) conjugated with anti-biotin antibodies to detect dual labeled amplicons, and anti-FITC/DIG antibodies to capture and immobilize the amplicons on the test-lines for visual indication (Fig 2). This technique eliminates the requirement for operator intervention. Moreover, the simplicity is also projected through the concise display of NALF results, using only two test lines; T1 for RIF resistance indication in the *rpoB* assay or INH resistance indication in the *katG* assay, and T2 for Mtb DNA control (Fig 2).

This NALF design contrasts other commercially existing DNA strip tests for MDR-TB such as GenotypeMTBDR*plus* which has 27 reaction zones on a single test strip, with 21 DNA probes for mutation detection and 6 probes as control [8]. All 27 lines representing *rpoB*, *katG* and *inhA* are displayed on the same test strip and have to be individually interpreted by the operator. The detailed and complex nature of the results renders it more suitable for epidemiological rather than diagnostic work. In comparison, the NALF device has no technical specification or interference requirements, making it more appropriate for diagnostics.

Another important advantage of using anti-tag antibodies on NALF rather than DNA probes is that it permits the indirect capture of target amplicons without necessitating DNA hybridization. This advantage makes the device generic, allowing broader application. The device can, thus, be adapted for the diagnosis of any disease requiring nucleic acid detection, based on the strict use of primers conjugated with specific tags; biotin for detection and FITC/DIG for capture (Fig 2). This generic nature allows the device to be mass produced at low cost, serving the objective of targeting low-resource settings. This design differs from the lateral flow designs adopted in previous studies in the determination of HIV and malaria infection where sequence specific oligonucleotide probes were used to capture HIV-RNA and *P*. *falciparum*-DNA [54–57].

As for primer design, we initially reasoned that the standard design of incorporating a single 3’-terminal mismatch to avoid primer hybridization with non-complementary DNA may be inadequate for the determination of single-nucleotide polymorphisms (SNPs). For this study, incorrect hybridization could lead to the misidentification of Mtb drug resistance, and therefore, MDR-TB. To minimize the possibility, the study explored the Yaku-Bonczyk primer design strategy entailing an additional intended mismatch to enhance specificity [15,53]. The study compared the functionality of the SM (single mutation) and DM (double mutation) primers (Table 2, Fig 7) to evaluate their performance in the selection for the *rpoB* assay. The criterion was based on specificity, efficacy, stringency and also their technical influence on the design of the NALF device. DM primers, with an additional strategic mismatch (Fig 6), were predicted to perform with a higher discrimination effect based on their success in other studies [15,53,58]. The results, however, defeated the expectations wherein the DM primers failed to anneal to their targets, providing false negative results. In this study, the DM primers were 15–17 bases in length. Comparatively, the primers employed in a previous study, used as guidance in the design of DM primers, is about 38 bases in length [15]. This distinction could have been the root cause of the second mismatch considerably weakening the annealing bond between the DM primers and their complementary MT template, resulting in complete non-binding [59]. SM primers, on the other hand, with the standard design of a 3’-terminal base complementing the MT templates (Fig 6), selectively annealed to their targets, resulting in a successful identification of the drug resistant (MT) and the drug susceptible (WT) Mtb isolates (Fig 7). The *rpoB* SM primers were, therefore, selected for use in multiplex PCR.

From the broad compilation of the global MDR-TB epidemiological statistics (Fig 4), 85–95% of RIF resistance was found to be accompanied by INH resistance; therefore, RIF resistance is popularly used by several existing commercial tests, such as XpertMTB/RIF and INNO-LiPA, as the surrogate marker for MDR-TB [43,46,60]. However, many studies point towards the benefit of testing for both RIF and INH resistance to improve the comprehensiveness of the results [38,61–63]. Retrospective studies have claimed that RIF monoresistance is on the rise in South-Africa, and testing for RIF resistance alone has led to the misidentification of MDR-TB [61,64]. Additionally, the incorporation of an INH assay would also support the determination of INH monoresistance. A recent study in China showed that early diagnosis of INH monoresistance allowed for the tailoring and implementation of specific therapies to prevent the development of MDR-TB [52]. The results of the study showed positive treatment outcomes for both patients diagnosed early with INH monoresistance as well as the drug-susceptible TB patients. A similar study conducted in the United States in 2009 also presented with comparable results [49]. INH resistance, however, is characterized by mutations in several genes (Fig 4) and aiming to detect all these genes would undesirably increase the complexity of the PCR-NALF test, based on the current design. This study, therefore, selected to focus only on the S315T mutation detection of the *katG* gene, an INH resistance conferring mutation with the highest global prevalence [29–34].

The current PCR-NALF test also presents an additional benefit of choice by dividing *rpoB* and *katG* into two separate assays. The option offers convenience. This design contrasts the test feature of GenotypeMTBDR*plus* where multiple probes for *rpoB*, *katG* and *inhA* are all pre-lined on one single test strip [8]. *RpoB* and *katG* assays in this study also share the same PCR thermocyling conditions, permitting the PCR amplification of both assays at the same time, if the user chooses to. The same test design could also be adapted for the determination of resistance to second-line drugs for the indication of extensively drug-resistant tuberculosis (XDR-TB), such as for the detection of fluoroquinolone(s) resistance, which is caused by single base mutations in the *gyrA* and *gyrB* genes [65,66]. The addition would add to the choice, as well as the coverage of the test.

Another important factor defining the validity of a molecular test is the least limit of detection (LOD), which signifies the sensitivity of the test. The LOD of the current test, for both the *rpoB* and *katG* assays, was found to be 10^4^ genomic copies per PCR reaction (0.1 ng of DNA) (Fig 9) using NALF. Even though the least LOD is relatively high, suggesting the need for further optimization, the current result of 10^4^ genomic copies could potentially be considered within the clinically relevant range. Sputum samples from TB patients with positive AFB staining of 1+ and 2+, based on the standard manual for laboratory technicians, is estimated to contain approximately 1–3x10^4^ and 3–5x10^4^ Mtb bacilli per ml of sputum, respectively [67]. Moreover, further studies using sputum samples are underway to help define the sensitivity and specificity of the test.

In spite of the promising results of the study, several aspects could still be improved. The LOD, which reflects the analytical sensitivity of the PCR-NALF test, could further be reduced through additional optimization, to enable a feasible detection of lower DNA concentrations. The extent of *rpoB* mutation coverage in the study could also be widened by incorporating the detection of more codons such as codons 533, 522, 513 and 511 within the rifampicin-resistance determining region [27,28,44,47]. In addition, further incorporation of *inhA* and *ahpC* genes, which account for 20–40% of INH resistance (Fig 4), could also improve the overall comprehensiveness of the test results.

In conclusion, this proof of concept study demonstrates the potential use of PCR-NALF as a molecular diagnostic alternative for the detection of MDR-TB. The simplicity, rapidity and ease-of-use could prove beneficial for low-resource settings that already employ PCR machines. Furthermore, the generic nature of the NALF device provides great diagnostic potential in adapting the test for the detection of other infectious and non-infectious diseases that require nucleic acid identification.
